# Preferential Interactions and the Effect of Protein PEGylation

**DOI:** 10.1371/journal.pone.0133584

**Published:** 2015-07-31

**Authors:** Louise Stenstrup Holm, Peter W. Thulstrup, Marina R. Kasimova, Marco van de Weert

**Affiliations:** 1 Department of Pharmacy, Faculty of Health and Medical Sciences, University of Copenhagen, Copenhagen, Denmark; 2 Department of Chemistry, Faculty of Science, University of Copenhagen, Copenhagen, Denmark; Universidad de Granada, SPAIN

## Abstract

**Background:**

PEGylation is a strategy used by the pharmaceutical industry to prolong systemic circulation of protein drugs, whereas formulation excipients are used for stabilization of proteins during storage. Here we investigate the role of PEGylation in protein stabilization by formulation excipients that preferentially interact with the protein.

**Methodology/Principal Findings:**

The model protein hen egg white lysozyme was doubly PEGylated on two lysines with 5 kDa linear PEGs (mPEG-succinimidyl valerate, MW 5000) and studied in the absence and presence of preferentially excluded sucrose and preferentially bound guanine hydrochloride. Structural characterization by far- and near-UV circular dichroism spectroscopy was supplemented by investigation of protein thermal stability with the use of differential scanning calorimetry, far and near-UV circular dichroism and fluorescence spectroscopy. It was found that PEGylated lysozyme was stabilized by the preferentially excluded excipient and destabilized by the preferentially bound excipient in a similar manner as lysozyme. However, compared to lysozyme in all cases the melting transition was lower by up to a few degrees and the calorimetric melting enthalpy was decreased to half the value for PEGylated lysozyme. The ratio between calorimetric and van’t Hoff enthalpy suggests that our PEGylated lysozyme is a dimer.

**Conclusion/Significance:**

The PEGylated model protein displayed similar stability responses to the addition of preferentially active excipients. This suggests that formulation principles using preferentially interacting excipients are similar for PEGylated and non-PEGylated proteins.

## Introduction

Next-generation protein drugs are proteins with altered amino acid sequence or altered glycosylation patterns, or proteins that are covalently modified with chemical moieties such as polyethylene glycol (PEG). These modifications are generally aimed at improving the pharmacokinetics of the protein, most commonly an increase in circulation half-life. In the case of PEGylation inert PEG chains are covalently conjugated to the protein, which can then circulate more than 20 times longer than the non-modified product depending on various protein- and modification specific characteristics. PEGylation of proteins has led to significantly improved possibilities for drug administration; for example, in treatment of chronic hepatitis C a 7-fold increase in circulation half-life is observed upon PEGylation of the native protein drug [1]. This allows once-weekly injections with improved clinical outcome compared to the thrice-weekly injections of the unmodified drug, despite the fact that receptor binding is reduced by more than a factor 10 for the PEGylated product [2]. Currently, there are 10 PEGylated proteins on the market [3].

The chemical aspects of the PEGylation process are well-documented, focusing on the different types of the PEGs, the coupling chemistry, the number of modifications and the targeting of different modification sites [4–8]. Also pharmacokinetic studies showing sustained plasma concentrations are common [9–12].

Comparatively fewer studies are available on the physical stability of PEGylated proteins. Those studies generally show a lower propensity for aggregation upon PEGylation, as shown for several proteins [6, 13–15]. Somewhat surprisingly, adsorption to hydrophobic surfaces is not reduced [13, 16]. The impact of PEGylation on thermal stability is less equivocal, with both decreases [13, 15] and increases [17] reported.

Considering the commercial success of protein PEGylation, there is surprisingly limited literature on pharmaceutical processing and formulation aspects. A few studies on processing by freeze-drying show that PEGylation improves stability [18–21]. While there are numerous studies on protein formulation approaches to achieve long-term storage stability (reviewed in [22–24]) proper formulation principles for PEGylated proteins are largely unreported in the scientific literature although it has undoubtedly been explored by the industry. It is therefore still unknown whether PEGylation may change the interactions between the pharmaceutical protein and commonly used excipients. Therefore, we have investigated the interactions between a PEGylated model protein and model excipients which are either preferentially excluded or bound. The model protein was lysozyme (Lyz), doubly PEGylated with 5 kDa units (LyzPEG). The preferentially excluded excipient was sucrose, which is present in various pharmaceutical formulations, and the preferentially bound excipient was guanidine hydrochloride (GdnHCl), which is commonly used to denature proteins. It is our hypothesis that PEGylation modifies the preferential interactions because PEG itself is preferentially active [25, 26]. The impact of the excipients was investigated in terms of structural stability by far- and near-UV circular dichroism (CD), while thermal stability was characterized by thermal denaturation using DSC, near- and far-UV CD and fluorescence. Furthermore, we discuss the spatial implications and possible interactions of PEG with the protein and possible explanations for the observed results.

## Materials

Hen egg white lysozyme dry powder (>95%), HEPES, guanidine hydrochloride and sucrose were purchased from Sigma. 5000 Da mPEG-succinimidyl valerate (mPEG-SVA)was purchased from Laysan Bio A/S.

## Experimentals

### PEGylation

Lysozyme was diPEGylated with a 5 kDa mPEG-SVA onto lysine residues and purified with IEC as described previously [13]. The diPEGylated species was used for all experiments, the main modification sites being Lys-33 and Lys-97 [27, 28] and to a minor degree Lys-116 [29] in the Lyz sequence.

### Sample preparation

1.67 M sucrose and 2.77 M GdnHCl stock solutions with 10 mM HEPES pH 7.4 were prepared and frozen until use. In our experience sucrose solutions may behave differently after being frozen or kept at 5°C prior to use at room temperature. Therefore, both excipient solutions were heated to 50–60°C to ensure homogeneity and then left to cool to room temperature before further use. Excipient concentrations were determined by refractometry. The refractive index was measured on a RL3 refractometer (Nr. 28046/01, PZO Warszawa, Warsaw, Poland). 5 measurements were averaged and a buffer average subtracted. The difference in the refractive index, *Δn*, is linear with the concentration of sucrose [30]. The concentration of GdnHCl concentration was calculated with the polynomial presented by Nozaki [31].

Protein stock solutions in 10 mM HEPES buffer pH 7.4 were diluted into the excipient solutions giving final excipient concentrations of 1.0 M sucrose and 2.0 M GdnHCl. At this concentration sucrose imparts a significant stabilization through preferential exclusion [32, 33]. A concentration of 2.0 M GdnHCl was chosen because for lysozyme it is well below the denaturing level [34, 35]. These excipient concentrations were used in all experiments. The protein concentrations were 0.1–2.5 mg/ml depending on technique. LyzPEG was measured in protein equivalent weight concentration. Concentrations were measured on a NanoDrop-1000 or a NanoDrop-2000c Spectrophotometer (Thermo Scientific, Wilmington, Delaware). Solutions were filtered through a 0.22 μm filter prior to DSC, CD and Fluorescence measurements.

### CD

Far- and near-UV circular dichroism spectroscopy was used for two purposes: structural assessment of Lyz and LyzPEG in buffer and in presence of excipients, and to follow temperature induced unfolding of the secondary and tertiary structure of the protein. Measurements were performed on degassed samples using a Jasco-815 CD instrument (JASCO, Essex, UK). The concentration was re-measured post degassing.

For recording of isothermal CD spectra for the structural assessment the instrument settings were: 0.1 nm data interval, 1 nm band width, digital integration time (DIT) of 4 s and 20 nm/min scan speed. 3 spectra were averaged, smoothed using a 25 point Savitzky-Golay algorithm (2^nd^ order), a buffer scan subtracted and the data were normalized to mean residue ellipticity (MRE) using a molecular weight of 14306 Da. Specifically, spectra of far-UV CD were collected at 2.5 mg/ml in a 50 μm circular quartz cuvette temperature controlled with a water bath set at 20°C. The spectra were recorded in the range 250–195 nm but are only displayed in the range 250–203 nm where the high tension always remained below 500 V. Near-UV CD were measured at a concentration of 0.4 mg/ml in a 10 mm quartz cuvette which was thermostated at 20°C using a Peltier element. The samples were recorded in the interval 320–240 nm and the maximum high tension at 280 nm was less than 420 V in all cases.

Temperature induced unfolding was investigated by analysis of the melting curves in both the far- and near-UV CD region. Samples were measured using rectangular quartz cuvettes and Peltier temperature control at a protein concentration of 0.5 mg/ml using a 1°C/min ramp rate and a data interval of 1°C in the temperature range 20–96°C. The heating rate used here is common for thermal unfolding analysis of proteins using DSC (see below).

The instrument parameters were set to 16 s digital integration time and the band width to 2 nm. For the temperature scans a 75 mm focusing lens was introduced in front of the sample, yielding a light spot of approximately 1 mm x 4 mm on the sample. The relevant buffer scans were subtracted and subsequently the data were fitted to a folded ↔ unfolded model in Microsoft Excel to obtain the temperature and the enthalpy of unfolding. Specifically, far-UV CD melting curves were measured at 222 nm for change in backbone structure (α-helical content) using a 1 mm cuvette (300 μl, max high tension < 530 V). Near-UV CD melting curves were measured at 257 nm and at 288.5 nm corresponding to the chiral activity bands of Phe and Trp side chains. Near-UV CD was measured using a 10 mm cuvette (1000 μl, maximum high tension < 330 V).

### DSC

Differential scanning calorimetry (DSC) was conducted on a NanoDSC (cell volume 299 μl, TA Instruments, Lindon, Utah) at 1 mg/ml of protein concentration. Heating was performed at 1°C/min in the temperature range from 20°C to 95°C. This heating rate is commonly used for thermal unfolding analysis of proteins in general [36] and lysozyme in particular [37–41]. Due to material restrictions we could not determine whether this scan rate was slow enough to allow sufficient time for the LyzPEG unfolding reaction to equilibrate or perform a reversibility assessment. Buffer scans were run until full overlay of two consecutive scans was obtained. Samples were degassed for 10 minutes prior to loading the solutions into the cells. Buffer subtraction, baseline correction and data treatment (non-2-state model) were conducted with OriginPro 8.6 (OriginLab, Northampton, Massachusetts, USA).

### Fluorescence

Fluorescence spectra were recorded as a function of temperature on a Spex Fluorolog 3–22 fluorescence spectrometer (Jobin-Yvon Horiba, Longjumeau, France) equipped with a 450 W xenon lamp. 1 ml of 0.1 mg/ml protein were placed in a 10 mm quartz cuvette, covered with a lid and stirred. Samples were excited at 295 nm and emission recorded from 300 nm to 450 nm with an increment of 0.5 nm. Excitation and emission slits were set to 1 and 3 nm, respectively. The data acquisition time was 0.1 s and 5 spectra were recorded and averaged at every degree from 20°C to 96°C. The temperature was controlled by water bath circulation and the temperature was measured directly in the water bath. Between each temperature increase the equilibration time was 1 minute and the tolerance for initializing the measurement was ± 0.5°C. Buffer scans were subtracted from the technical spectra (uncorrected for instrument characteristics) and the data were smoothed with a 25 point Savitzky-Golay algorithm. Maximum peak analyses were performed by fitting the curves to a Gaussian function around the apparent peak maximum. Due to unexpected spectral fluctuations for the sucrose-containing solutions the spectrum of Lyz in sucrose was fitted to a Gaussian function of the whole spectrum. The transition midpoint temperature (T_m_) and enthalpy of unfolding (ΔH) were determined by fitting the peak maxima (λ_max_) as a function of temperature to a 2-state model in Microsoft Excel. GraphPad Prism 5.03 for Windows, GraphPad Software, San Diego, California, USA was used for the Gaussian fit and graph presentation.

### Structural images

The structure of Lyz was represented with PyMOL (The PyMOL Molecular Graphics System, Version 1.7.2.3 Schrödinger, LLC) using the pdb-entry 1E8L of an NMR based solution structure of hen egg white lysozyme [42] to visualize the most probable PEGylation sites as well as the protein residues relevant for evaluation of the experimental data. PoPMuSiC 2.0 [43] was used to calculate the exposure of the tryptophan residues.

## Results

### Secondary and tertiary structure

**Fig 1:**
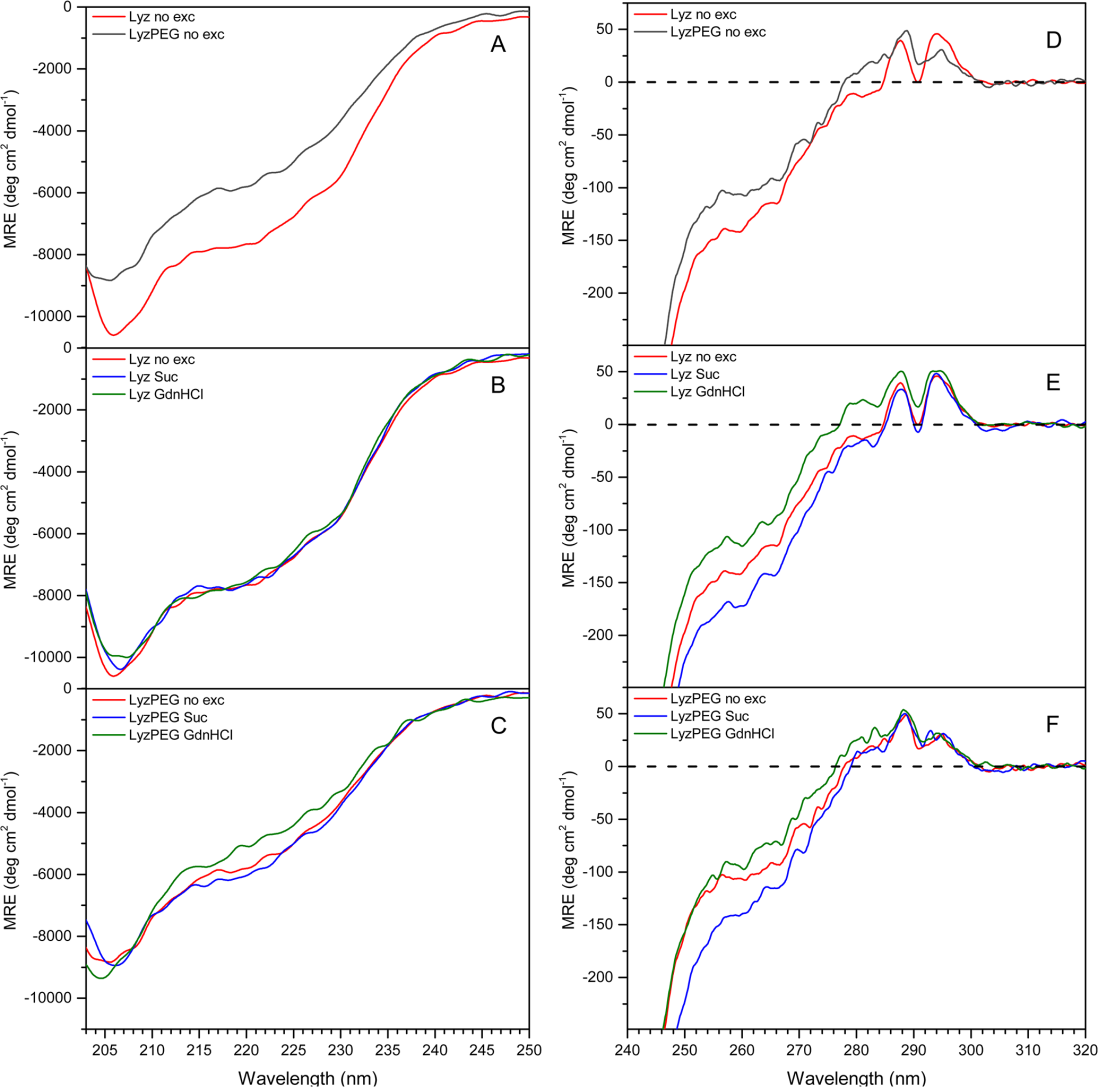
Far- and near- UV CD spectra measured at 20°C, pH 7.4 in HEPES buffer. Excipients are 1.0 M sucrose and 2.0 M GdnHCl. (A-C) Far-UV CD spectra of (A) Lyz and LyzPEG without excipients, (B) Lyz with excipients and (C) LyzPEG with excipients; (D-F) near-UV CD spectra of (D) Lyz and LyzPEG without excipients, (E) Lyz with excipients and (F) LyzPEG with excipients.

The impact of the PEGylation process and the effect of the two model excipients on the structural characteristics of Lyz was determined using far- and near-UV spectroscopy. The far-UV CD spectra show that LyzPEG (Fig 1A) has a lower (85% at 205 nm) signal at the same molar concentration as Lyz indicating a change in the secondary structure compared to the non-PEGylated, native protein [44]. For both proteins addition of 1.0 M sucrose does not alter the secondary structure (Fig 1B and 1C). Addition of 2.0 M GdnHCl has a marginal effect on Lyz (Fig 1B) and a stronger effect on LyzPEG (Fig 1C). The spectral changes are primarily observed in the region 203–235 nm, indicative of a minor loss of α-helical content.

In the near-UV region, the absorbance is dominated by contributions from the 6 Trp residues, and also includes dichroic signals of the 3 Tyr, 3 Phe and the 4 disulphide groups. Assignment of the CD signals in this region is highly complex, as the chiral environment of the chromophore plays an important role in determining magnitude and direction of the signal. Furthermore, due to the large number of absorbing species there is a high probability of sign cancellation. The near-UV CD spectra of the native and PEGylated protein (Fig 1D) show a clear fine structure with 2 peaks at 300–275 nm at positive ellipticities attributed to Trp signals, and a shoulder around 265–250 nm at negative ellipticities often attributed to Phe signals [45]. The Lyz fine structure at positive ellipticities consists of two strong bands at 294 nm and 286 nm of equal intensity and a weaker fine structure at 278 nm. The fine structure of LyzPEG is slightly different with a diminished signal at 294 nm and a peak shift from 286 nm to 288 nm. For Lyz the shoulder has a delicate fine structure with 2 small positive peaks at 257 nm and 264 nm which has been observed earlier in similar solution conditions [46]. For LyzPEG only the fine structure at 257 nm remains. The LyzPEG CD signal at 257 nm is reduced compared to Lyz, similar to the signal reduction observed in the far-UV range.

For Lyz (Fig 1E) addition of sucrose does not change the Trp spectral features, but the Phe band gains in negative intensity confirming some type of reorganization and possible stabilization. Addition of GdnHCl reduces the signal in the full range of the spectrum. The stabilization of LyzPEG in presence of sucrose (Fig 1F) is only visible at the shoulder at 257 nm, and the response to sucrose is similar to that seen for native Lyz. GdnHCl reduces the signal of LyzPEG but to a lesser extent than for Lyz, which is opposite to the observations for the far-UV CD.

### Thermal denaturation

Thermal denaturation of Lyz and LyzPEG with and without excipients was followed by DSC, far-UV CD at 222 nm and near-UV CD at 257 nm (Phe) and 288.5 nm (Trp).

**Fig 2:**
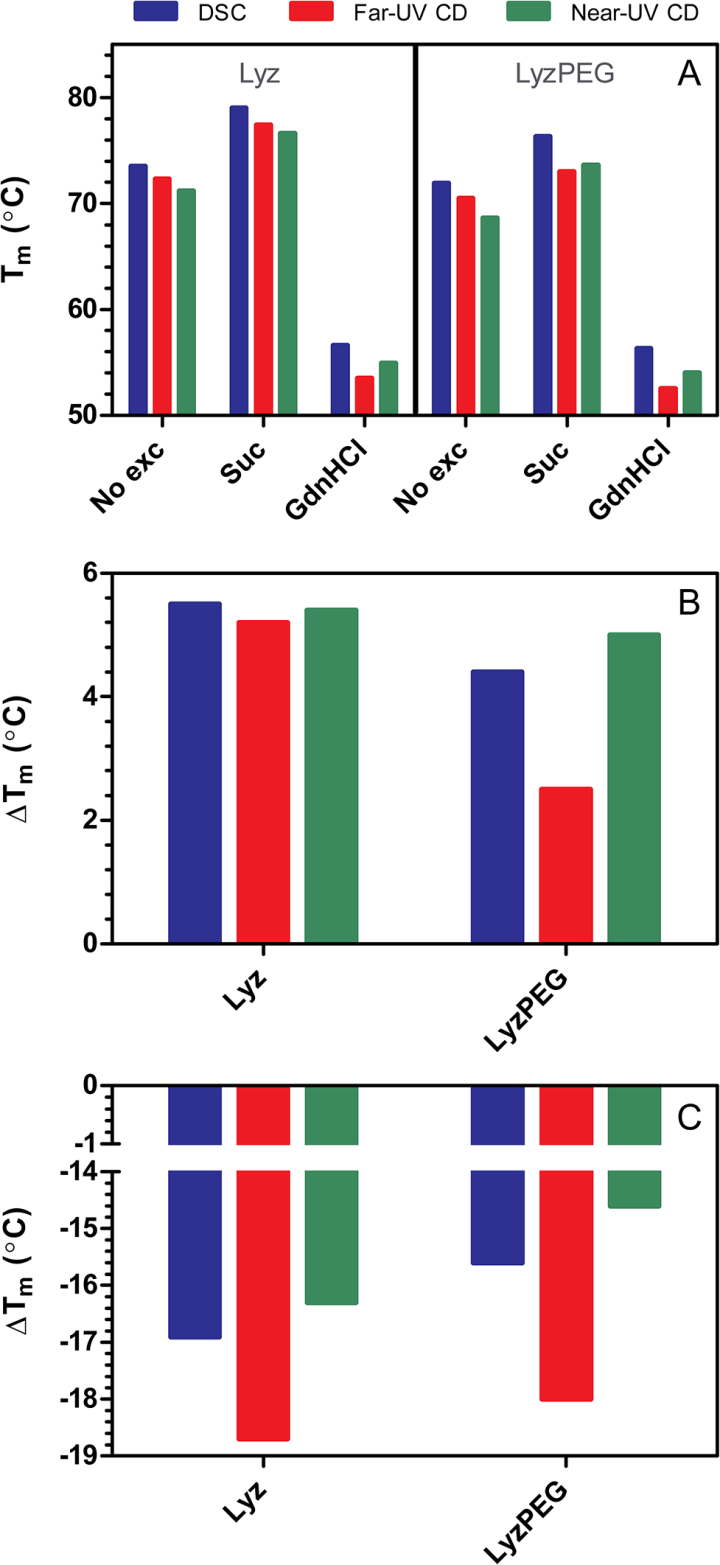
Melting transition temperatures and changes as a function of excipient presence. Blue: DSC non-2-state fit, red: far-UV CD at 222 nm (α-helical content) and green: near-UV CD global fit of 257 nm (Phe signal) and 288.5 nm (Trp signal). The figures show A) melting transition temperatures. B) Sucrose induced change in melting transition temperatures. C) GdnHCl induced change in melting transition temperatures.

The DSC thermograms were fitted to a non-2-state model after subtraction of a cubic baseline. Lyz fit well to a 2-state model, while LyzPEG did not. A satisfactory fit of LyzPEG was obtained with a non-2-state model and as Lyz unfolding is reversible [47–49], reversibility was also assumed for LyzPEG. Material restrictions prevented reversibility assessment and elucidation of potential scan rate dependency and consequently the calculated thermodynamic parameters must be taken with due caution and are only meant for qualitative comparisons. For consistency and comparability both Lyz and LyzPEG were fitted to a non-2-state model. Similar values were obtained for Lyz using the 2 models and a simple analysis of area under the curve (corresponding to the calorimetric enthalpy) and apparent melting temperature (T_max_ instead of T_m_) for all samples gave similar values (S2 Table) to the ones obtained with the non-2-state model. The obtained values for T_m_ and enthalpies are represented in Fig 2 and Table 1 (T_m_ values in S1 Table). Although the T_m_ values are often used as an indication of protein thermodynamic stability at room temperature [50–52], the correct approach would be to compare Gibbs free energy (ΔG) of the two proteins at physiologically relevant temperatures. This requires knowledge of the heat capacity change upon unfolding (ΔC_P_), which can be obtained directly from the DSC thermograms [53]. Unfortunately, this was not possible in our case, due to uncertainty in drawing the post-denaturational baselines, which were affected by the post-denaturational aggregation of lysozyme [47]. However, an estimate of the ΔC_P_ can be made from the mutual dependence between ΔH_cal_ and T_m_ (S5 Fig). The CD results were fitted to a simple folding-unfolding model in Excel as described in the literature [54]. The near-UV CD data at 257 nm and 288.5 nm were fitted globally as well as individually and the results of the individual fits are available in S3 Table. The LyzPEG CD data fit equally well to a dimer model (S3 Fig, see also discussion below). In all cases the heat capacity change was fixed to 0 as the fitting quality was not improved upon introduction of non-zero ΔC_p_. Melting curves and their respective fits can be found in the S1 Fig and S2 Fig, the resulting T_m_ and enthalpies are shown in Fig 2 and Table 1.

**Table 1:** Enthalpy of unfolding from various techniques.

	Method	DSC (non-2-state)			Far-UV CD	Near-UV CD	Fl λ_max_
	Parameter	ΔH_cal_ (kJ/mol)	ΔH_vH_ (kJ/mol)	ΔH_vH_/ΔH_cal_	ΔH (kJ/mol)	ΔH (kJ/mol)	ΔH (kJ/mol)
Lyz	No excipients	405	481	1.2	404	562	442
	Suc	481	472	1.0	510	553	458
	GdnHCl	306	363	1.2	314	380	400
LyzPEG	No excipients	175	372	2.1	248	286	-
	Suc	156	398	2.5	167	348	-
	GdnHCl	112	294	2.6	239	276	-

The CD and fluorescence derived enthalpies correspond to the DSC derived van’t Hoff enthalpies. Near-UV CD is a global fit of 257 nm and 288.5 nm. Fluorescence peak maximum revealed that Lyz in sucrose had an additional transition at 50°C with ΔH = 495 kJ/mol.

### DSC

The DSC data provided an estimate for the T_m_ value for Lyz at 73.5°C and 1.6°C lower for LyzPEG at 71.9°C. In the presence of sucrose the T_m_ of Lyz is shifted to 79.0°C, an increase of 5.5°C, and in the presence of GdnHCl the T_m_ is decreased by 16.9°C to 56.6°C. LyzPEG shows similar melting temperature shifts as Lyz in response to the addition of excipients, although all melting temperatures are lower than those for Lyz. In sucrose LyzPEG has a T_m_ of 76.3°C, which is a stabilization of 4.4°C, and GdnHCl lowers the LyzPEG T_m_ to 56.3°C, which is a decrease of 15.6°C. The transition midpoint temperatures are presented graphically in Fig 2A and the differences in T_m_ values as a function of sucrose and GdnHCl are presented in Fig 2B and 2C, respectively.

The calorimetric melting enthalpy (ΔH_cal_) of Lyz is 405 kJ/mol which corresponds reasonably well with previous studies [55]. For LyzPEG, however, the calorimetric enthalpy is less than half (175 kJ/mol) of that value, while the ratio of van‘t Hoff enthalpy (ΔH_vH_) to calorimetric enthalpy ΔH_cal_ is ca. two, suggesting that LyzPEG unfolds as a dimer. For Lyz the ΔH_cal_ and ΔH_vH_, were essentially the same consistent with Lyz being a monomer. All ΔH_cal_, ΔH_vH_ values and ΔH_vH_/ΔH_cal_ ratios are summarized in Table 1. With ΔH_cal_ = 481 kJ/mol sucrose clearly stabilizes Lyz, but for LyzPEG the addition of sucrose decreases ΔH_cal_ to 156 kJ/mol. The denaturant GdnHCl reduces the calorimetric melting enthalpy to 306 kJ/mol and 112 kJ/mol for Lyz and LyzPEG, respectively, which means both proteins are destabilized to the same extent. The ΔH_vH_/ΔH_cal_ ratios suggest that LyzPEG stays dimeric in the presence of both excipients.

T_m_ and ΔH_cal_ obtained from the DSC data in all three solution conditions were used to calculate the heat capacity change upon unfolding, ΔC_p_, of Lyz and LyzPEG (S5 Fig). This approach gives ΔC_p_ of 7.35 kJ/(K*mol) and 2.68 kJ/(K*mol) for the Lyz and LyzPEG, respectively. The ΔC_p_ for Lyz compares well with earlier studies [39, 56]. The ΔC_p_ for LyzPEG is lower by more than a factor 2, which is in agreement with the decreased overall structure of the PEGylated protein observed by CD. The heat capacity change upon unfolding was then used to estimate the Gibbs free energy function for both proteins using the modified Gibbs-Helmholtz equation, and as anticipated the LyzPEG was less stable at all temperatures between 0°C and T_m_ compared to Lyz. At room temperature the ΔG value of LyzPEG was about half of that of the native Lyz (S5 Fig).

### CD

Thermal denaturation studies by CD show similar trends as the DSC analysis (Fig 2 and Table 1). For the far-UV CD melting the T_m_ values are ca. 1–4°C lower than those measured by DSC for both proteins and in all solution conditions. In sucrose the T_m_ of Lyz increases by 5.1°C, similar to that observed by DSC, and the LyzPEG T_m_ increases by 2.5°C, almost half of that observed by DSC. In the presence of GdnHCl the far-UV CD T_m_ is decreased ca. 2°C further for both proteins compared to DSC.

In near-UV CD the changes for both Lyz and LyzPEG also occur at a lower temperature than measured by DSC. The T_m_ values are also lower than in the far-UV CD experiments except in the presence of GdnHCl, where a slightly higher T_m_ is observed as compared to that determined by far-UV CD.

The melting enthalpy for Lyz determined from the far-UV CD is lower than that observed by DSC, while the melting enthalpy for Lyz in presence of both excipients agrees better with the DSC data (Table 1) under both solution conditions. ΔH for LyzPEG is also lower than expected when comparing to the DSC ΔH_vH_ value. The addition of sucrose to LyzPEG apparently decreases the melting enthalpy, whereas this remains almost constant in the DSC data. Upon addition of GdnHCl the LyzPEG far-UV CD melting enthalpy remains the same as in buffer, and is twice as high as the corresponding ΔH_cal_.

The melting enthalpies calculated from the near-UV CD are generally higher for both modified and unmodified proteins compared to DSC and far-UV CD. The only exception is that LyzPEG near-UV CD melting enthalpies are lower compared to the DSC van’t Hoff enthalpies. For Lyz the addition of sucrose does not change the enthalpy. Addition of GdnHCl reduces the enthalpy to the same extent as observed with DSC and far-UV CD. The near-UV CD enthalpy for LyzPEG in buffer is half that of Lyz. It is a bit higher than the far-UV CD enthalpy, much higher than the ΔH_cal_, but lower than the ΔH_vH_. Excipients exert a different effect on LyzPEG compared to Lyz: for LyzPEG the enthalpy is greatly increased by addition of sucrose, whereas addition of GdnHCl has no effect. The latter was also observed with far-UV CD. S1 Text contains a more elaborate discussion on data quality, including explanations for the apparent discrepancies in the enthalpy values obtained by the different techniques.

### Fluorescence

**Fig 3:**
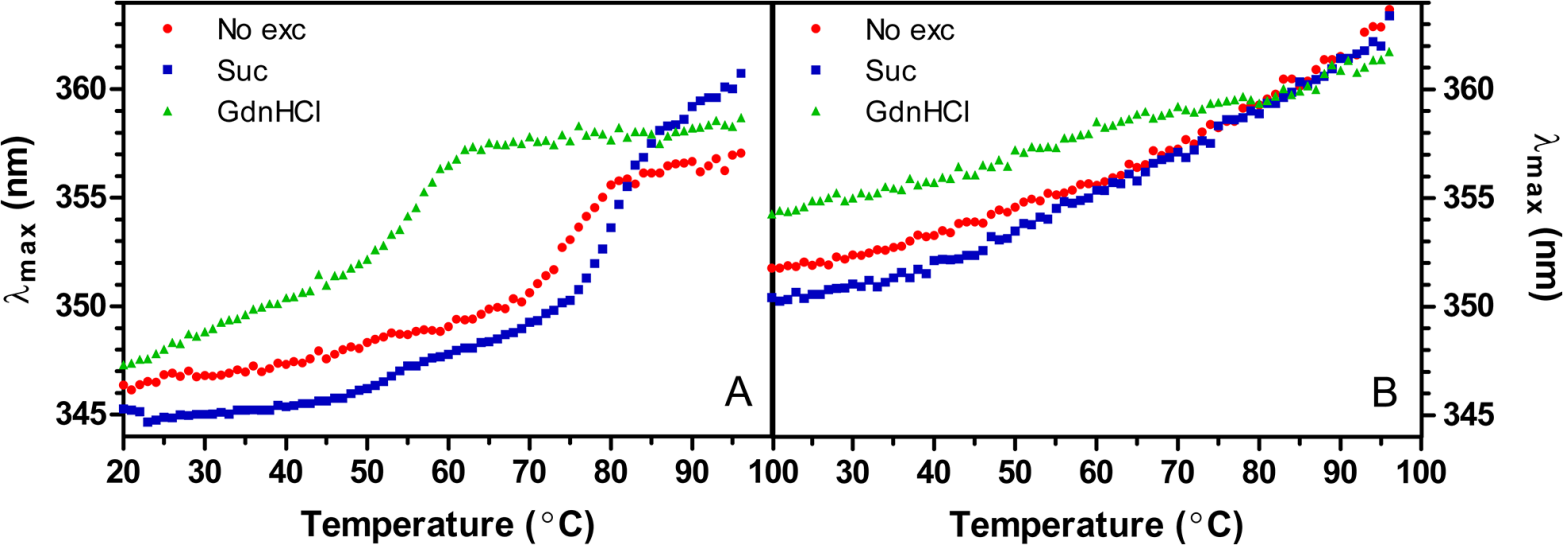
Peak maximum of fluorescence spectra as function of temperature and excipient. A) Lyz B) LyzPEG.

Characterization of the temperature-induced unfolding was also attempted using fluorescence spectroscopy. Lyz displays a clear melting transition (Fig 3A) while LyzPEG merely shows a gradual redshift during heating (Fig 3B) which does not allow calculation of the denaturation parameters. The ΔH (Table 1) and T_m_ values of Lyz correspond well with the calorimetric data. The melting points of Lyz are 75.2°C, 79.7°C and 56.6°C for no excipient, sucrose and GdnHCl containing solutions, respectively. An additional minor transition at 50°C is observed for Lyz in sucrose.

The fluorescence traces of the LyzPEG solutions lack clear melting transitions and the λ_max_ values are consistently red shifted by 5–7 nm throughout the whole temperature range compared to Lyz, indicating an increased solvent exposure of the active tryptophans.

## Discussion

### Structural features exploited in lysozyme

**Fig 4:**
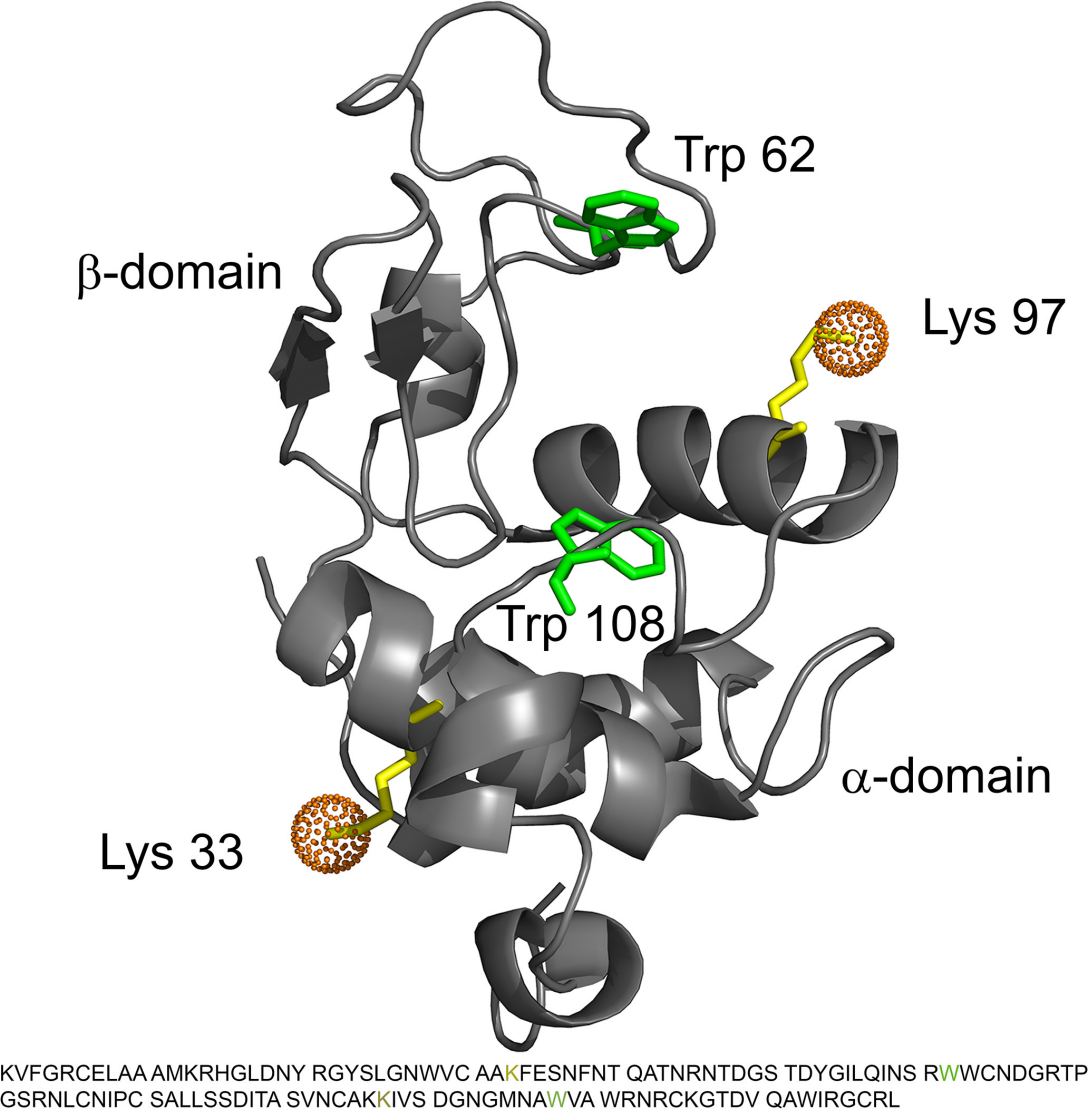
Ribbon structure of hen egg white lysozyme (pdb entry 1E8L). The PEGylated lysines are indicated in yellow and the fluorescence active tryptophans are indicated in green. The sequence of lysozyme is given below the structure with the same color indications for the mentioned lysines and tryptophans.

Lysozyme is a very-well characterized protein and the 3D structure has been determined both in solution by NMR and in crystal form by protein X-ray crystallography. Fig 4 depicts the structure of Lyz from Schwalbe *et al*. [42], with the two lysines that are most likely PEGylated marked in yellow. The fold of the protein is characterized by two sub-domains, α and β. The α-domain consists of a 3_10_-helix and 4 α-helices (res. no. 1–35 and 85–129), and the β-domain is composed of a short triple-stranded antiparallel β-sheet, a loop and a 3_10_-helix (res. no. 36–84). The α-domain is stabilized by a hydrophobic core. Additionally, 4 disulphide bonds play an important role for the stabilization of the tertiary and secondary structure [57]. There is a general consensus that Lyz unfolds in a 2-state unfolding process [58, 59] although some studies have suggested a more complicated unfolding process at low pH [53]. Of spectroscopic relevance there are 6 Trp and 3 Tyr and 3 Phe units in the native protein. The bulk of Lyz fluorescence is attributed only to Trp residues 62 and 108 (Fig 4, marked in green) [60]. The accessible surface area (ASA) calculated by PoPMuSiC shows that in the native state the side chain of Trp-62 is somewhat solvent-exposed (ASA = 42%) while Trp-108 is buried (ASA = 5.7%). Emission from the other Trp units is likely quenched by nearby cystine groups [60, 61].

### Effect of PEGylation

All analytical techniques used in the current study indicate that our PEGylated lysozyme has an altered conformation and stability compared to the native lysozyme, which corresponds to our earlier finding [13]. The far- and near-UV CD spectra indicate a loss of α-helical structure in PEGylated protein and some local changes around the Trp residues. As depicted in Fig 4 both PEG-chains are attached to α-helical motifs which may destabilize the implicated helices resulting in a reduced α-helix signal. A reduced far-UV CD signal has been observed before for LyzPEG monoPEGylated at Lys-33 [28].

The thermal denaturation studies show both a reduced thermal stability of LyzPEG, indicated by a lower melting temperature, and a reduced thermodynamic stability, with the ΔG_u_ value at 25°C being about half of that found for Lyz, suggesting that the LyzPEG is not fully folded. The latter is reflected in the lower enthalpy of denaturation at the T_m_ and the diminished Gibbs free energy function in the temperature range of 0°C—T_m_ (S5 Fig). While denaturation of the native lysozyme is consistent with the 2-state model, the DSC melting profile of LyzPEG can only be fitted to the non-2-state model, indicating a change in the denaturation mechanism of LyzPEG. Unfortunately, material limitations prevented further elucidation of the unfolding mechanism and the potential contribution of kinetically controlled events. This introduces an uncertainty in the validity of the thermodynamic analysis, which in turn means the results can only be used for qualitative comparative purposes. The ratio of van‘t Hoff to calorimetric enthalpy of 2:1 (Table 1) suggests that the PEGylated protein forms a dimer. At the same time, the total calorimetric enthalpy of LyzPEG is lower, indicating that the energy of dimerization is insufficient for compensating the enthalpy loss from the partial unfolding. The dimeric nature of LyzPEG is supported by the small-angle X-ray scattering studies reported in our previous publication [13]. However, fitting the melting curves of LyzPEG to a model where the protein unfolding is coupled to dimer dissociation was unsuccessful, failing to reproduce the sharp shape of the LyzPEG denaturation transition. Inability of the dimer-denaturation model to properly describe DSC data could be due to the fact that this model does not take into account the partially denatured starting state of LyzPEG. In addition, it is unclear to what extent the potential interference from the PEG moiety could affect the shape of the DSC profile. In contrast, the dimer-denaturation model described the CD data very well, indicating that the spectroscopic techniques were insensitive to the selection of the fitting model as both monomer and dimer fitting results were of equally good quality.

The fluorescence spectroscopic analysis of LyzPEG spectra shows a significant red-shift of the peak maximum suggesting increased solvent exposure of the fluorescent tryptophans. This is another indication of the partially denatured initial state of LyzPEG with a consequent exposure of Trp residues that are normally buried in the native Lyz. In addition, the absence of a clear transition in the LyzPEG fluorescence melting data suggests that the local environment around the tryptophans is already disrupted at room temperature, so that increasing temperatures lead only to the gradual shifts in Trp fluorescence properties.

Another potential explanation for the altered fluorescent properties of LyzPEG is the presence of PEG. It is unlikely that the dimer formation causes a quenching of the two active tryptophans by cysteine groups of the neighboring protein. Such a quenching would have reduced the overall fluorescence intensity, but the fluorescence intensity was found to be constant within the concentration uncertainties for all solutions (S4 Fig). Furness *et al*. found that the cleft between the α- and β-domain is a binding site for free 4 kDa PEG [62]. Both Trp-62 and Trp-108 are positioned close to the cleft and thus close proximity to PEG could shield or interfere with the spectroscopic changes during unfolding.

### Effect of sucrose

**Fig 5:**
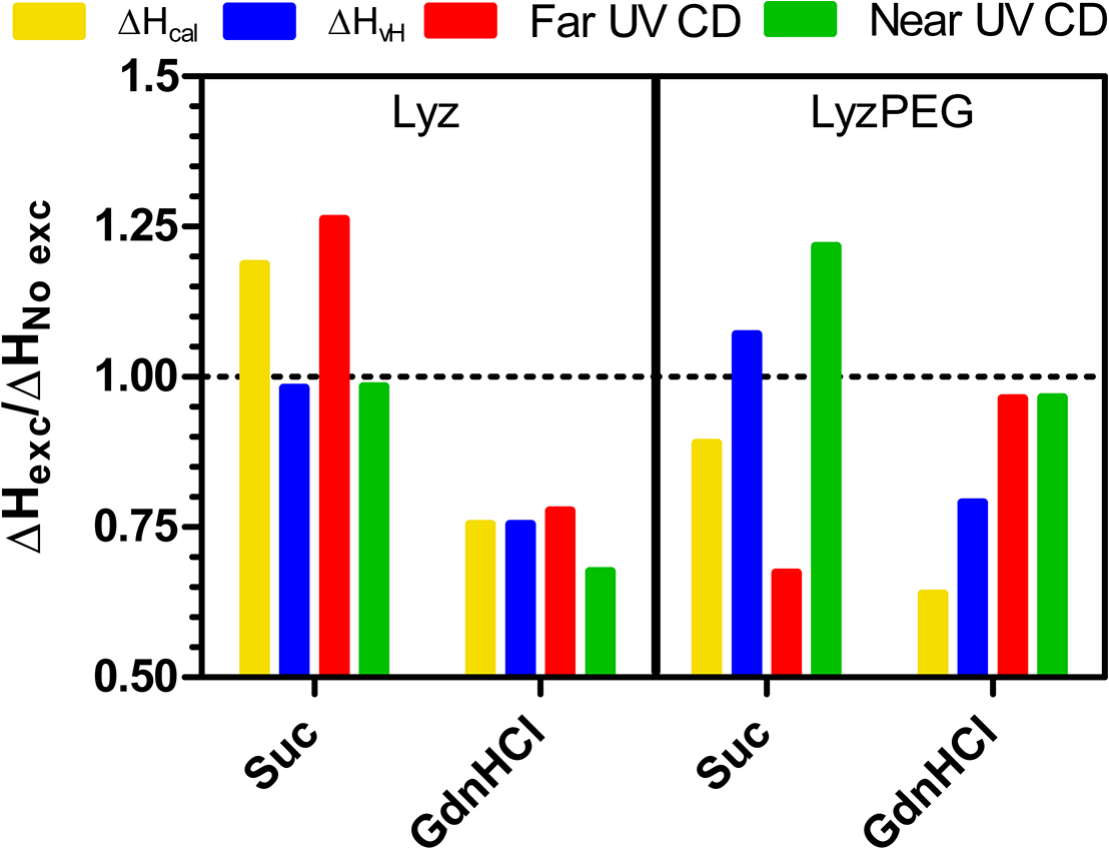
Relative change in enthalpy of unfolding upon excipient addition. Yellow: DSC calorimetric enthalpy from non-2-state fit, Blue: DSC van’t Hoff enthalpy from non-2-state fit, red: far-UV CD at 222 nm (α-helical content) and green: near-UV CD global fit of 257 nm (Phe signal) and 288.5 nm (Trp signal). The LyzPEG ratios are calculated based on lower absolute values (see Table **1**) likely with a larger impact of fitting uncertainty and thus resulting in an apparent larger variation.

Addition of the preferentially excluded excipient sucrose to Lyz and LyzPEG has no impact on the secondary structure, and a minor effect on the tertiary structure of the protein, as indicated by far- and near-UV CD spectra, respectively (Fig 1). This minor effect is not unexpected, as the preferential exclusion of sucrose is known to reduce the structural flexibility of a protein [63] without affecting the secondary structure [64]. As also expected, the addition of sucrose resulted in increased T_m_ values [32, 40, 65]. This increase (Fig 2B), 5–6°C for Lyz, and 2.5–5°C for LyzPEG, is observed in all techniques used. The large variability of the change in T_m_ for LyzPEG is unexpected, with the T_m_ determined by far-UV CD (222 nm) responsible for the low extreme. We have no unequivocal explanation for this variability, but it may be due to the dimeric nature of the LyzPEG and the associated complex unfolding process. The effect of sucrose on the LyzPEG appears to have the largest impact on the tertiary fold around some Phe residues, as suggested by the relatively large change in the near-UV CD spectrum around 257 nm. The relative change in van’t Hoff enthalpy as a result of excipients (Fig 5) is fairly constant for Lyz with no ΔH_vH_ change in the sucrose solution, except in far-UV where it is higher.

The changes in enthalpy of unfolding for LyzPEG are more variable (Fig 5), with a slightly increased van’t Hoff enthalpy, a strong decrease for the far-UV data and an increase for the near-UV data. This variability could be due to the lower transition enthalpies of the LyzPEG and concomitant larger noise levels that impacted the fitting. As noted earlier, an increase for the unfolding enthalpy is expected upon addition of a preferentially excluded excipient [33, 66], and in principle this should be observed irrespective of the protein folding characteristic that is being followed. In summary, our data do not show any unequivocal differences of the impact of sucrose on the thermal stability between Lyz and LyzPEG.

### Effect of GdnHCl

Lyz denaturation by GdnHCl is known to be pH dependent. At room temperature and neutral pH Lyz starts to unfold at GdnHCl concentrations around 3 M [34, 35], at pH 4–6 it is denatured above 2 M [67–70] and 2 M is sufficient to start denaturing Lyz below pH 4 [49, 71]. Thus, at the pH used in this study (7.4) no significant unfolding is expected. This is confirmed by the far- and near-UV CD (Fig 1), which show no change in the Lyz secondary structure, minor changes in the Lyz tertiary structure, and also minor changes in the secondary and tertiary structure of LyzPEG. As anticipated, GdnHCl does have a destabilizing effect, as shown by the significant reduction in unfolding temperature. In our case the Lyz T_m_ is decreased by ca. 16.5–18.5°C and the LyzPEG T_m_ is decreased a bit less by 14.5–18.0°C (Fig 2C). As expected upon a decrease in melting temperature, the unfolding enthalpy is reduced by a fourth for Lyz in the presence of GdnHCl (Fig 5) in both DSC, far- and near-UV CD. For LyzPEG this is not the case. A similar reduction is observed in ΔH_vH_, but there are no changes for the far-UV or near-UV CD analyses. The DSC results suggest that LyzPEG responds similarly to GdnHCl as Lyz. While there are some inconsistencies between CD spectral data and CD thermal denaturation data both for far- and near-UV CD on the effect of GdnHCl, these are small. In conclusion, GdnHCl also does not cause any major difference in behavior of Lyz versus LyzPEG.

## Conclusion

Overall, our results show that within the limits of the different methods PEGylation of lysozyme has no, or a minor, impact on the preferential interaction with our model excipients. The preferential exclusion of sucrose and preferential binding of GdnHCl are somewhat lower for LyzPEG, as shown primarily by the change in melting temperature, but this may well be a result of the altered folding of LyzPEG caused by the PEGylation itself. Thus, the thermodynamic stabilization and destabilization of PEGylated proteins by preferentially active excipients is likely similar to that observed for the unmodified protein. This then means that PEGylated proteins can be stabilized using the same preferential interaction formulation principles as used for non-PEGylated proteins. Further experiments including multiple proteins should be performed to support this conclusion.

## Supporting Information

S1 Fig DSC results and fitting. The first row shows raw data (insert: protein and buffer) as well as the data with buffer subtracted and the cubic baseline to be subtracted before fitting. The second row shows area under the curve (AUC) which is comparable with the enthalpy (only this number is based on the true data whereas **Δ**H is based on the fit of the data). The 3^rd^ row shows the simplest fitting model: a 2-state fit (data in black, fit in red). The fit is very poor for LyzPEG in GdnHCl and these values were therefore not used. The 4^th^ row shows the fit to a non-2-state model and the T_m_ values are listed in S2 Table. It was not possible to fit LyzPEG in presence of sucrose to a 2-state model.
(PDF)

S2 Fig CD melting curves (buffer subtracted) and fitting to a 2-state model where ΔC_p_ = 0. 1^st^ row shows far-UV CD melting at 222 nm corresponding to the change in secondary structure (especially α-helix content). The 2^nd^ row shows near-UV CD melting at 257 nm corresponding to the phenylalanine signal. The 3^rd^ row shows near-UV CD melting at 288.5 nm corresponding to the Trp fine structure. The 4^th^ row shows the global fit of the two near-UV CD data sets. The T_m_-values for the far-UV and global fit of near-UV data are presented in S1 Table. The T_m_ and ΔH values for the individual near-UV fit are presented in S3 Table.
(PDF)

S3 Fig Global fit of near-UV CD data (257 nm and 288.5 nm) of LyzPEG to a dimer model. A) no excipients B) 1.0 M sucrose C) 2.0 M GdnHCl. For the simple monomer unfolding model the transition midpoint temperature (T_m_) coincides with the temperature, where the change in Gibbs free energy is equal to 0 (T_ΔG = 0_). In case of the dimer unfolding model the fitted T_ΔG = 0_ values are higher than the T_m_ values [57]. However, inspection of the fit indicates that population of the denatured state becomes 50% at the temperatures close to the T_m_ values calculated from the monomer unfolding model.
(PDF)

S4 Fig Fluorescence spectra of Lyz and LyzPEG in HEPES buffer pH 7.4 at 20°C. The graphs demonstrate the apparent red-shift in LyzPEG whereas the fluorescence intensity remains the same.
(PDF)

S5 Fig Gibbs free energy function, calculated with the help of ΔCp values obtained from the slope of ΔHcal vs Tm dependence. The insert shows the thermodynamic parameters used for ΔCp determination. ΔG was calculated using the modified Gibbs-Helmholtz equation as described in *Vaz DC, Rodrigues JR, Sebald W, Dobson CM, Brito RMM. Enthalpic and entropic contributions mediate the role of disulfide bonds on the conformational stability of interleukin-4. Protein Sci. 2006;15(1):33–44*.
(PNG)

S1 Table Melting transition temperatures from DSC and CD with Lyz and LyzPEG in various solution conditions. Values are presented graphically in Fig 2A in the article.
(DOCX)

S2 Table DSC results of values obtained with different models. Integration was performed using a linear baseline while the 2-state and non-2-state were fitted using a cubic baseline as seen in S1 Fig. Integration results are AUC which is comparable to ΔH and T_max_ which is comparable to T_m_. Lyz fitted well to a 2-state model and are included here. Values from the non-2-state fit presented in the article are included here for comparison.
(DOCX)

S3 Table Near-UV CD 2-state fit of single data sets recorded at 257 and 288.5 nm. (DOCX)

S1 Text CD data quality (DOCX)
