# Supplementary material for: Preferential Interactions and the Effect of Protein PEGylation
Source: PLoS One. 2015 Jul 31;10(7):e0133584. doi: 10.1371/journal.pone.0133584 (PMC4521882; doi:10.1371/journal.pone.0133584)
Supplement: S1 Fig — The first row shows raw data (insert: protein and buffer) as well as the data with buffer subtracted and the cubic baseline to be subtracted before fitting. The second row shows area under the curve (AUC) which is comparable with the enthalpy (only this number is based on the true data whereas ΔH is based on the fit of the data). The 3rd row shows the simplest fitting model: a 2-state fit (data in black, fit in red). The fit is very poor for LyzPEG in GdnHCl and these values were therefore not used. The 4th row shows the fit to a non-2-state model and the Tm values are listed in S2 Table. It was not possible to fit LyzPEG in presence of sucrose to a 2-state model. (PDF) [file pone.0133584.s001.pdf]

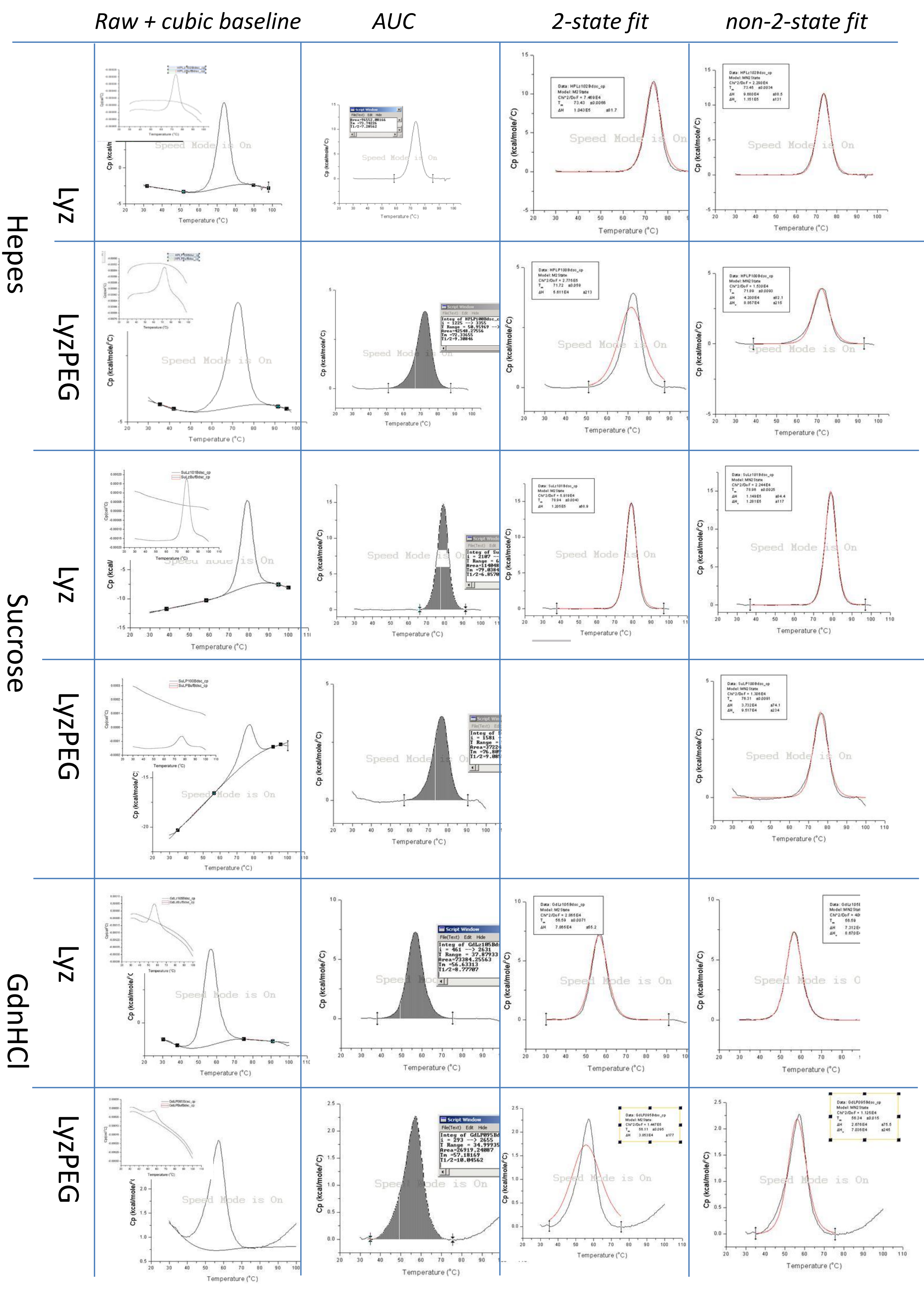

Raw + cubic baseline
AUC
2-state fit
non-2-state fit
Hepes
Lyz
LyzPEG
Sucrose
Lyz
LyzPEG
GdnHCl
Lyz
LyzPEG
Temperature (°C)
Cp (kcal/mole/°C)
