# Supplementary figures and images for: Preferential Interactions and the Effect of Protein PEGylation
Source: PLoS One. 2015 Jul 31;10(7):e0133584. doi: 10.1371/journal.pone.0133584 (PMC4521882; doi:10.1371/journal.pone.0133584)

Hepes

Lyz

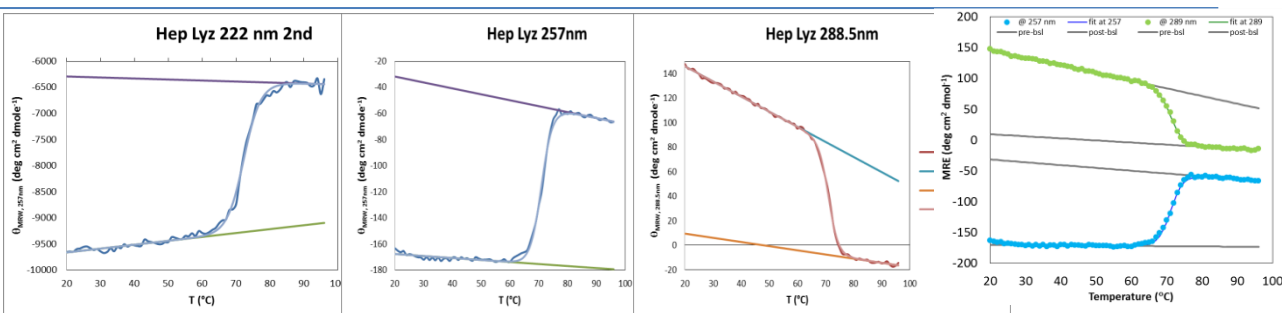

LYZPEG

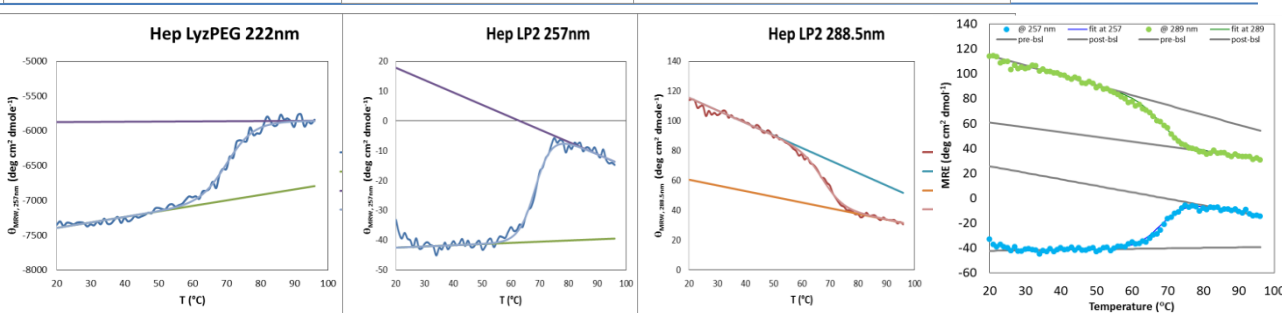

Sucrose

Lyz

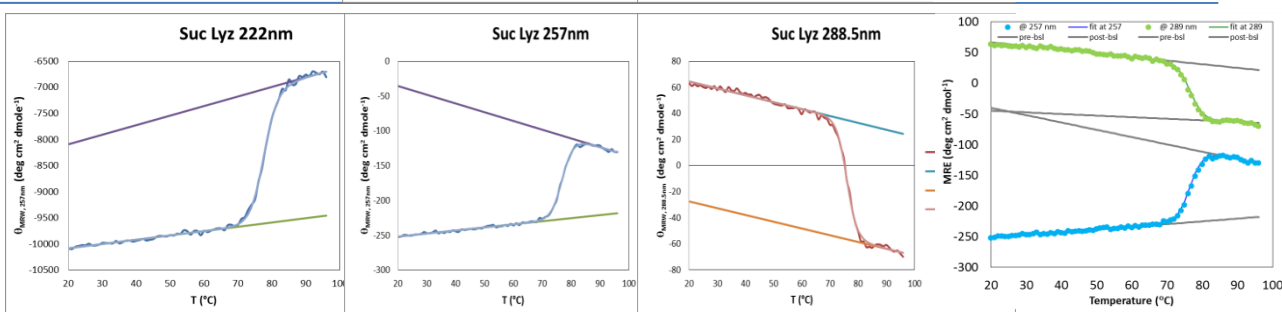

LYZPEG

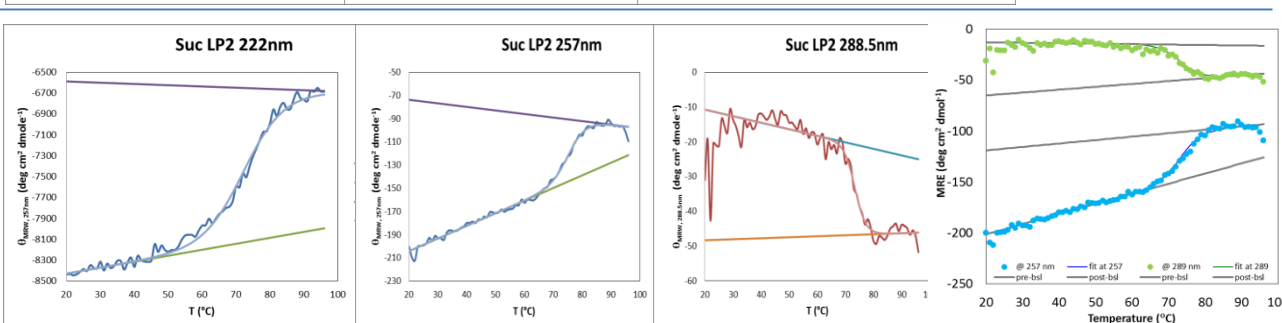

GdnHCl

Lyz

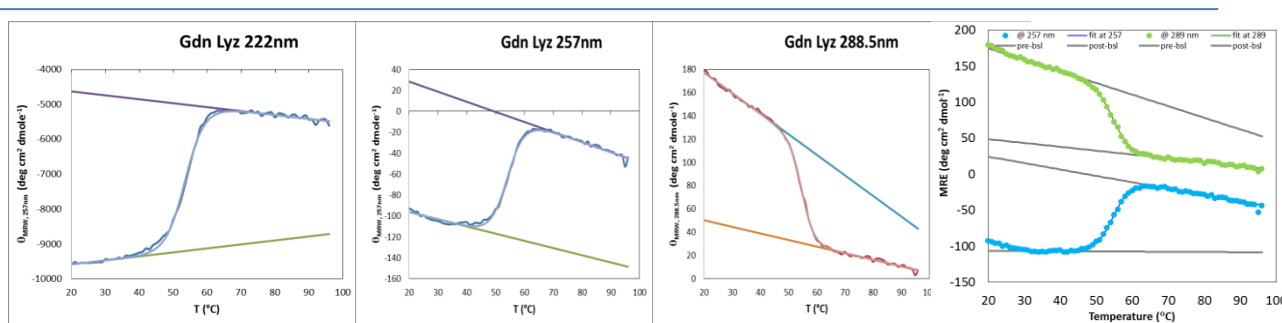

LYZPEG

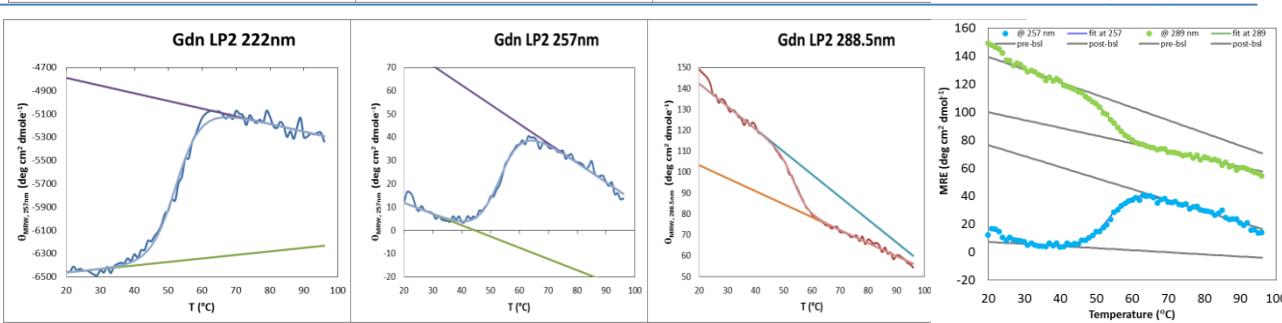

Supplement: S2 Fig — 1st row shows far-UV CD melting at 222 nm corresponding to the change in secondary structure (especially α-helix content). The 2nd row shows near-UV CD melting at 257 nm corresponding to the phenylalanine signal. The 3rd row shows near-UV CD melting at 288.5 nm corresponding to the Trp fine structure. The 4th row shows the global fit of the two near-UV CD data sets. The Tm-values for the far-UV and global fit of near-UV data are presented in S1 Table. The Tm and ΔH values for the individual near-UV fit are presented in S3 Table. (PDF) [file pone.0133584.s002.pdf]

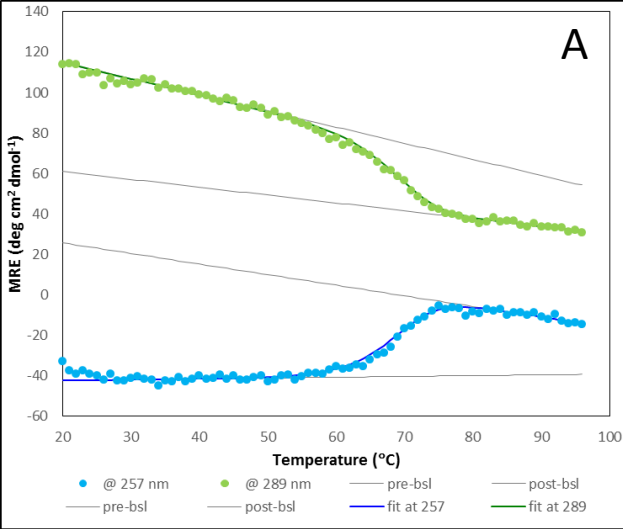

| LyzPEG        | $\Delta H$ (kJ/mol) | $T_{\Delta G=0}$ (°C) |
|---------------|---------------------|-----------------------|
| No excipients | 426                 | 94.1                  |
| Sucrose       | 448                 | 99.1                  |
| GdnHCl        | 413                 | 78.0                  |

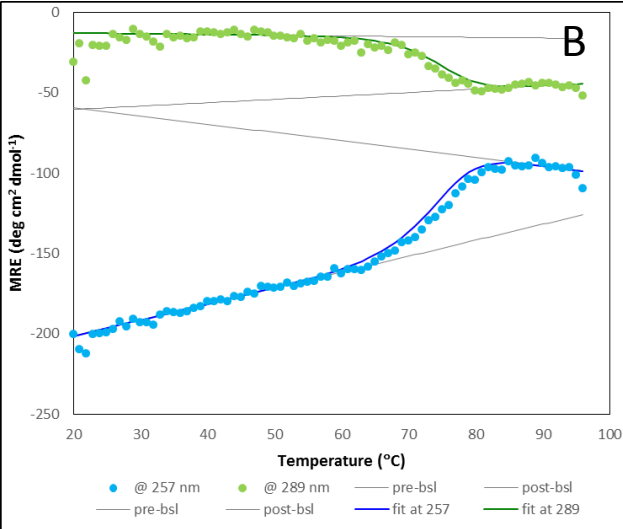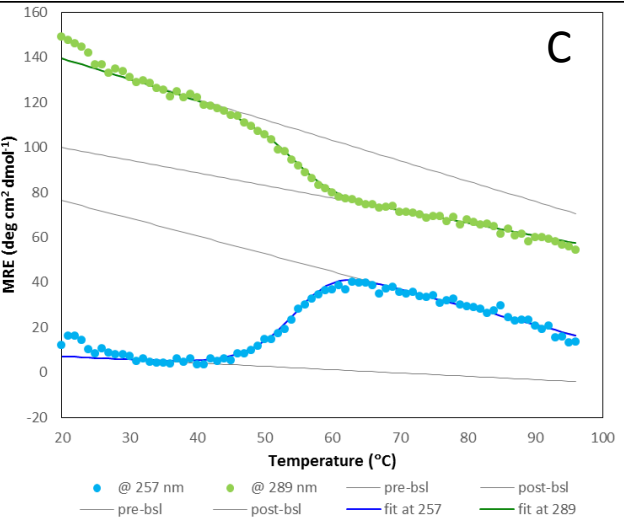

Supplement: S3 Fig — A) no excipients B) 1.0 M sucrose C) 2.0 M GdnHCl. For the simple monomer unfolding model the transition midpoint temperature (Tm) coincides with the temperature, where the change in Gibbs free energy is equal to 0 (TΔG = 0). In case of the dimer unfolding model the fitted TΔG = 0 values are higher than the Tm values [57]. However, inspection of the fit indicates that population of the denatured state becomes 50% at the temperatures close to the Tm values calculated from the monomer unfolding model. (PDF) [file pone.0133584.s003.pdf]

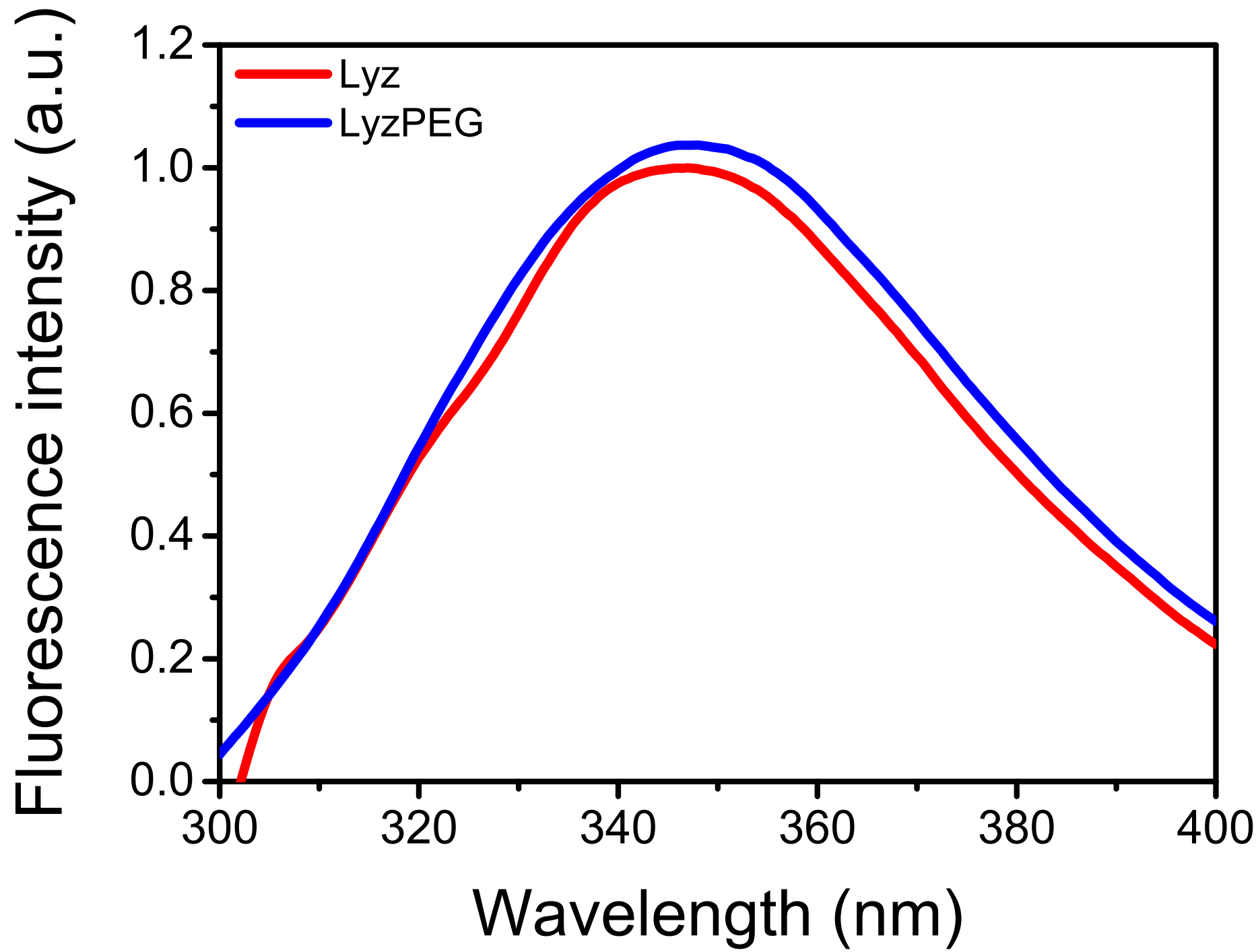

Supplement: S4 Fig — The graphs demonstrate the apparent red-shift in LyzPEG whereas the fluorescence intensity remains the same. (PDF) [file pone.0133584.s004.pdf]

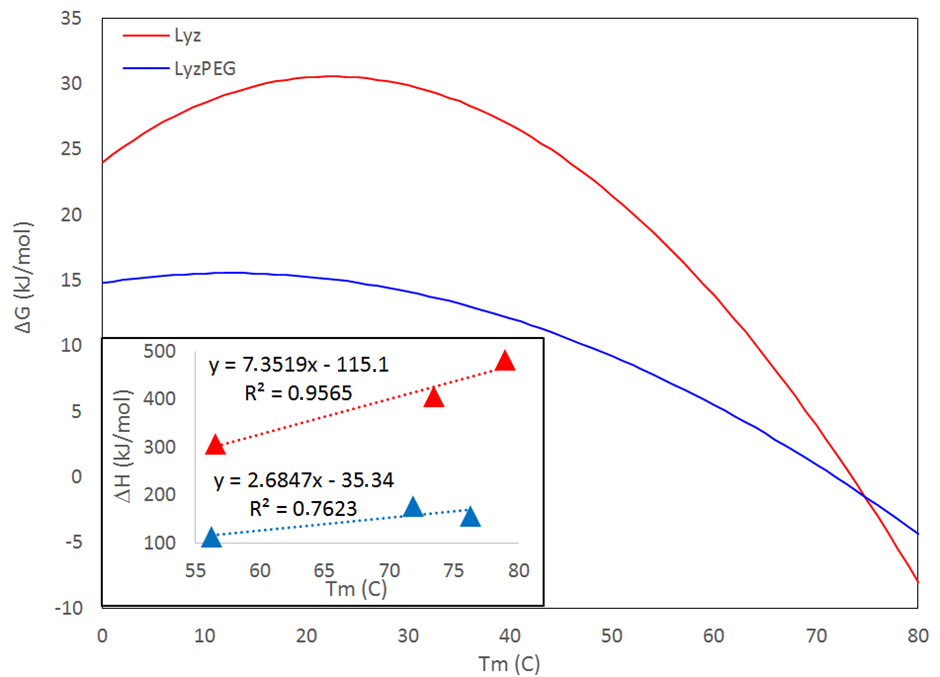

Supplement: S5 Fig — The insert shows the thermodynamic parameters used for ΔCp determination. ΔG was calculated using the modified Gibbs-Helmholtz equation as described in Vaz DC, Rodrigues JR, Sebald W, Dobson CM, Brito RMM. Enthalpic and entropic contributions mediate the role of disulfide bonds on the conformational stability of interleukin-4. Protein Sci. 2006;15(1):33–44. (PNG) [file pone.0133584.s005.png]
