# Supplementary material for: Preferential Interactions and the Effect of Protein PEGylation
Source: PLoS One. 2015 Jul 31;10(7):e0133584. doi: 10.1371/journal.pone.0133584 (PMC4521882; doi:10.1371/journal.pone.0133584)
Supplement: S1 Table — Values are presented graphically in Fig 2A in the article. (DOCX) [file pone.0133584.s006.docx]

|  |  |  | DSC  (non-2-state) | Far-UV CD 222 nm | Near-UV CD Global fit |
| --- | --- | --- | --- | --- | --- |
| T_m_ (°C) | Lyz | No excipients | 73.5 | 72.3 | 71.2 |
|  |  | Suc | 79.0 | 77.4 | 76.6 |
|  |  | GdnHCl | 56.6 | 53.5 | 54.9 |
|  | LyzPEG | No excipients | 71.9 | 70.5 | 68.6 |
|  |  | Suc | 76.3 | 73.0 | 73.6 |
|  |  | GdnHCl | 56.3 | 52.5 | 54.0 |
