# Supplementary material for: Preferential Interactions and the Effect of Protein PEGylation
Source: PLoS One. 2015 Jul 31;10(7):e0133584. doi: 10.1371/journal.pone.0133584 (PMC4521882; doi:10.1371/journal.pone.0133584)
Supplement: S2 Table — Integration was performed using a linear baseline while the 2-state and non-2-state were fitted using a cubic baseline as seen in S1 Fig. Integration results are AUC which is comparable to ΔH and Tmax which is comparable to Tm. Lyz fitted well to a 2-state model and are included here. Values from the non-2-state fit presented in the article are included here for comparison. (DOCX) [file pone.0133584.s007.docx]

|  |  | Integration | 2-state | non-2-state |
| --- | --- | --- | --- | --- |
|  |  | AUC (kJ/mol) | ΔH_cal_ (kJ/mol) | ΔH_cal_ (kJ/mol) |
| Lyz | No excipients | 404 | 436 | 405 |
|  | Suc | 477 | 504 | 481 |
|  | GdnHCl | 307 | 329 | 306 |
| LyzPEG | No excipients | 178 | - | 175 |
|  | Suc | 156 | - | 156 |
|  | GdnHCl | 113 | - | 112 |
|  |  | T_max_ (°C) | T_m_ (°C) | T_m_ (°C) |
| Lyz | No excipients | 73.7 | 73.4 | 73.5 |
|  | Suc | 79.0 | 78.9 | 79.0 |
|  | GdnHCl | 56.6 | 56.6 | 56.6 |
| LyzPEG | No excipients | 72.3 | - | 71.9 |
|  | Suc | 76.8 | - | 76.3 |
|  | GdnHCl | 57.2 | - | 56.3 |
