# Supplementary material for: Preferential Interactions and the Effect of Protein PEGylation
Source: PLoS One. 2015 Jul 31;10(7):e0133584. doi: 10.1371/journal.pone.0133584 (PMC4521882; doi:10.1371/journal.pone.0133584)
Supplement: S3 Table — (DOCX) [file pone.0133584.s008.docx]

|  |  | 257 nm | 288.5 nm |
| --- | --- | --- | --- |
|  |  | ΔH (kJ/mol) | ΔH (kJ/mol) |
| Lyz | No excipients | 514 | 576 |
|  | Suc | 533 | 565 |
|  | GdnHCl | 304 | 418 |
| LyzPEG | No excipients | 337 | 268 |
|  | Suc | 339 | 332 |
|  | GdnHCl | 223 | 351 |
|  |  | T_m_ (°C) | T_m_ (°C) |
| Lyz | No excipients | 70.8 | 71.1 |
|  | Suc | 76.9 | 75.9 |
|  | GdnHCl | 54.1 | 54.8 |
| LyzPEG | No excipients | 68.6 | 68.1 |
|  | Suc | 75.9 | 72.1 |
|  | GdnHCl | 53.4 | 54.0 |
