# Supplementary material for: Preferential Interactions and the Effect of Protein PEGylation
Source: PLoS One. 2015 Jul 31;10(7):e0133584. doi: 10.1371/journal.pone.0133584 (PMC4521882; doi:10.1371/journal.pone.0133584)
Supplement: S1 Text — (DOCX) [file pone.0133584.s009.docx]

## CD data quality

The fitting of both far- and near-UV CD data (see S2 Fig.) was complicated due to the limited data quality despite our efforts to obtain high quality data. The variability at low and high temperatures made it difficult to accurately estimate the pre- and post-baseline, respectively, which are important for the fitting of particularly the ΔH. This was especially apparent for the single fit of 288.5 nm data of LyzPEG. It was generally a lesser problem for the Lyz samples. LyzPEG in sucrose was the most difficult data to fit for two reasons: the transition broadening which is a common effect of PEGylation and the very short post-transition baseline as a result of the stabilizing nature of sucrose (transition midpoint at higher temperatures). Furthermore, the change in near-UV CD signal at 288.5 nm is very small which leads to a large noise level. The fitting errors due to baseline variations are within 60 kJ/mol.
